# Magnetic resonance angiography for cerebral arterial and venous visualization in dogs: a comparative study of time-of-flight, phase-contrast, and time-resolved imaging of contrast kinetics sequences

**DOI:** 10.3389/fvets.2025.1533130

**Published:** 2025-02-18

**Authors:** Sunghwa Hong, Soyeon Kim, Mire Namgoong, Minjoo Kwon, Junghee Yoon, Jihye Choi

**Affiliations:** ^1^Department of Veterinary Medical Imaging, College of Veterinary Medicine, Seoul National University, Seoul, Republic of Korea; ^2^NEL Animal Medical Center, Anyang-si, Republic of Korea

**Keywords:** dog, velocity encoding, canine, TWIST, VENC, MRI

## Abstract

This study aims to compare the effectiveness of three magnetic resonance angiography (MRA) sequences—Time-of-Flight (TOF), Phase-Contrast (PC), and Time-Resolved Imaging of Contrast Kinetics (TRICKS)—in visualizing cerebral arteries and veins in dogs. The goal is to determine the most suitable MRA sequence for improving diagnostic accuracy in canine cerebral imaging. Five healthy adult beagles underwent imaging using a 1.5-T MRI system. Quantitative measures, including signal-to-noise ratio (SNR) and contrast-to-noise ratio (CNR), were calculated, and qualitative evaluations of vessel delineation and visualization symmetry were performed by blinded observers. The three sequences were statistically compared using Friedman and Wilcoxon signed-rank tests. All dogs completed the imaging protocols without any complications. TOF MRA showed the best results for visualizing cerebral arteries, with the highest SNR and CNR in major arteries like the basilar, rostral, caudal cerebral, and caudal communicating arteries. TRICKS MRA performed better than TOF and PC for venous imaging, achieving higher SNR or CNR in most veins, including the transverse, temporal, straight sinus and dorsal cerebral vein, and had the shortest acquisition time. In qualitative evaluations, TOF MRA demonstrated acceptable or higher vascular visibility in most of brain arteries. All venous structures were significantly better visualized with TRICKS MRA compared to TOF MRA. Interobserver agreement was high, showing consistency in the results. These findings suggest that TOF is best for artery imaging, while TRICKS is ideal for venous imaging and faster procedures. TOF is recommended for clear and detailed imaging of cerebral arteries, while TRICKS is more suitable for imaging veins and situations where faster scans are needed. These results provide a basis for improving MRA techniques in veterinary medicine, helping to make diagnoses more accurate and procedures more efficient in both clinical practice and research.

## 1 Introduction

Magnetic resonance imaging (MRI) is the preferred diagnostic modality for evaluating brain tumors, as it provides high-resolution images of tumor characteristics such as necrosis, hemorrhage, and vascularity. However, in cases where tumors may displace or involve major cerebral vessels, magnetic resonance angiography (MRA) can be useful for evaluating vascular structures and planning precise surgical interventions. In particular, MRA allows for accurate delineation of the relationship of the tumor with surrounding vessels through detailed visualization of tumor-feeding arteries and adjacent critical vascular structures in highly vascularized tumors (1, 2). In human medicine, the detailed anatomical understanding of the arteries supplying the meningiomas is emphasized, because this information is not only vital for accurate diagnosis but also treatment decisions, leading to improved outcomes by targeting feeding vessels for surgical removal or therapeutic interventions. In addition, the evaluation of the intracranial circulation provides valuable information in the diagnosis and prognosis of vascular abnormalities, such as aneurysms, arterial and venous steno-occlusive diseases, inflammatory arterial diseases, and congenital vascular abnormalities in human medicine (3).

The arterial supply to the canine brain is primarily provided by the internal carotid and basilar arteries. The two arteries are connected by the rostral and caudal communicating arteries, forming the circle of Willis. From this circle of Willis, the cerebrum receives its blood from three major bilateral arteries: the rostral, middle, and caudal cerebral arteries. The rostral portion of the cerebellum is primarily supplied by the rostral cerebellar arteries, which typically originate from the caudal communicating artery (4). The superficial cerebral venous drainage is conducted by the dorsal cerebral veins that drain into the dorsal sagittal sinus. The dorsal sagittal sinus receives venous blood from the straight sinus and then bifurcates into the paired transverse sinuses. These transverse sinuses run laterally within the osseous canal, then divide into the sigmoid sinuses ventrally and the temporal sinuses (5, 6). High-resolution vascular imaging of these structures is crucial in understanding normal and pathological conditions in veterinary patients.

In brain vascular imaging, both computed tomography angiography (CTA) and MRA are used. While CTA offers faster imaging times and lower costs compared to MRA, CTA only provides lower soft tissue contrast resolution, and if the scan timing is not optimal, the insufficient enhancement of intracranial arteries or veins can reduce the diagnostic accuracy. In contrast, MRA can provide superior soft tissue contrast and does not rely on timing-dependent contrast enhancement. Importantly, MRA can be performed either with or without a contrast agent, allowing flexibility in diagnostic applications.

Among MRA techniques, time-of flight (TOF), phase-contrast (PC), contrast enhanced methods like time-resolved imaging of contrast kinetics (TRICKS) provide distinct advantages and limitations. TOF MRA sequences relies on differential amplitude of magnetization between flowing blood and stationary tissues. Protons entering the imaged slice produce strong signals due to their higher magnetization, while stationary protons have low magnetization and thus show suppressed signals due to successive excitation pulses that saturate their magnetization. Pre-saturation pulses placed adjacent to the imaging slice can be used to specifically image arteries or veins by suppressing the signal of flow in the given direction. In the veterinary medicine, TOF sequences allowed detailed visualization of major intracranial arteries, including the internal carotid artery, basilar artery, rostral communicating artery, caudal communicating artery, and the rostral, middle, and caudal cerebral arteries, using MRI systems with field strengths ranging from 1 T to 7 T (7–11). However, TOF is sensitive to flow-related artifacts and there is no study to evaluate the use of TOF sequences for MR venography in veterinary medicine.

PC MRA provides information on flow velocity and direction based on the different phase shift between stationary and moving spins by applying a velocity-encoding gradient (12). By adjusting the amplitude and duration of this gradient, blood flow at different speeds can be visualized, ranging from slow venous flow at 10 cm/s to fast arterial flow at 80 cm/s. However, PC MRA has limited application in veterinary brain study: only one study evaluated the anatomical variations in the dural venous sinus system using PC MRA (13).

TRICKS, a contrast-enhanced technique, improves temporal resolution by sampling central k-space more frequently and interpolating data to achieve continuous high-frequency updates, enabling a detailed view of both arterial and venous phases (14, 15). To the authors’ knowledge, no studies have applied TRICKS to brain angiography in veterinary medicine.

In human medicine, numerous studies have compared these different MRA techniques to optimize MRA protocols such as between 3D time-resolved MRA and conventional contrast-enhanced bolus chase MRA, as well as 3D TOF MRA, 3D contrast-enhanced elliptic centric-ordered MR venography versus TOF MR venography in the intracranial venous system, and TOF versus PC MR angiography (16–19). However, such studies are limited in veterinary medicine. As a result, it is unclear which MRA technique is the most effective for cerebral vascular imaging in dogs.

Therefore, this study applied TOF, PC, and TRICKS sequences to normal dogs and compared the visualization of cerebral arteries and veins to canine brain MRA. This study hypothesized that each major cerebral artery and vein could be optimally visualized with specific sequences, and that TRICKS would provide superior visualization across cerebral vessels compared to conventional MRA sequences. The aim of this study was to determine the most effective MRA sequence for diagnostic objectives in canine cerebral imaging, in order to improve diagnostic accuracy within a limited timeframe.

## 2 Materials and methods

This study was approved by the Institutional Animal Care and Use Committee of Seoul National University, and the animals were cared for in accordance with the Guidelines for Animal Experiments of Seoul National University (SNU-240717-5). Furthermore, this study adhered to the ARRIVE guidelines to ensure ethical and transparent reporting of animal experiments in line with international standards.

### 2.1 Animals

This experimental, prospective, pilot exploratory study included five adult purpose-bred beagles. The small sample size in this study can be attributed to several factors. First, the use of advanced imaging equipment such as MRI entails significant financial and time constraints. Second, as an exploratory pilot study, the aim was to evaluate the feasibility and effectiveness of the protocols rather than generalizing findings to a larger population. This study included three males and two females. The median age of the dogs was 3 years (Range: 2–4 years), and the mean weight was 10 kg (range: 8–11 kg). All dogs were clinically healthy based on physical examination, blood pressure measurement, complete blood count, biochemistry, urinalysis, thoracic and abdominal radiography, abdominal ultrasonography, and echocardiography. The dogs had no history or clinical signs of neurologic diseases. They had not been subjected to any prior invasive procedures. They were individually housed and fed commercial dry food and tap water ad libitum.

### 2.2 Anesthesia

After an 8-h fast, a 20-gage catheter was inserted into the cephalic vein and anesthesia was induced via intravenous injection of 0.1 mg/kg medetomidine (Domitor®) and 2.0 mg/kg alfaxalone (Alfaxan®, Jurox Pty Ltd., Rutherford, NSW, Australia) in each dog. Then, anesthesia was maintained with sevoflurane (Sevofran®, Hana Pharm, Korea) and oxygen (1–1.2 L/min) via an endotracheal tube. The dogs were maintained under positive pressure ventilation using 100% oxygen with a peak inspired pressure of 10–12 mmHg at a respiratory rate of 10–12 breaths/min, tidal volume of 15 mL/kg, and end-tidal CO_2_ concentration of 35–45 mmHg. During the anesthesia, heart rate, end-tidal carbon dioxide, body temperature, and noninvasive blood pressure were monitored. After completing the imaging procedures, anesthesia was discontinued, and the dogs were monitored closely during recovery. The endotracheal tube was removed once the palpebral reflex, gag reflex, and the ability to lift their head had returned. The dogs were discharged to the facility once they were fully alert and able to stand unassisted. During the recovery period, heart rate, respiratory rate, and body temperature were continuously monitored to ensure stability. Any potential adverse effects related to anesthesia were carefully assessed.

### 2.3 MRA protocols

Underwent general anesthesia, the dog was placed in the sternal recumbency positioned with the head first in the scanner. All MRI scans were performed using a 1.5-T magnet (SIGNA Creator; GE Healthcare, Milwaukee, WI, USA) with an 8-channel knee coil. Three orthogonal plane images of 14–16 cm of field of view (FOV) were obtained with T2-weighted MRI. Then, MRA of the variable FOV in range 15–28 cm were performed using transverse three dimensional (3D) TOF MRA for arteriogram and venogram, sagittal phase-contrast (PC) MRA for venogram, and dorsal 3D TRICKS sequences for arteriogram and venogram were performed sequentially.

The parameters used for 3D TOF MRA, 3D PC MRA, and TRICKS analyses are presented in Table 1. In 3D TOF arteriogram, a saturation pulse is placed rostral to the slab to null venous signals. Conversely, in 3D TOF venogram, it is placed caudal to the slab to null arterial signals. In PC MRA, the velocity-encoding gradient was set at 15 cm/s to facilitate visualization of slow-velocity venous flow. For TRICKS MRA, a single intravenous dose (0.2 mmol/kg) of a paramagnetic gadolinium-based contrast agent (Dotarem, Guerbet, France) was administered through a cephalic vein catheter using an automatic power injector (OptiStar® Elite, Mallinckrodt, Missouri, USA) at a rate of 2 mL/s, followed immediately with 10 mL of normal saline at the same rate. Time resolved imaging was performed over 164 s, and during the total scan time, 25 time frames were captured at 5.9 s intervals to visualize the movement of contrast agents within the blood vessels over time: The mask was acquired for 24 s before contrast injection as a baseline images, and after the contrast agent was injected, the temporal postcontrast acquisition lasted for 147.5 s, during which 25 separate images were captured (one every 5.9 s). The acquisition time for TRICKS was recorded from the start of post-contrast imaging to the end of the sequence, excluding the mask acquisition time. After subtraction processing using baseline images, 3D reconstructed MRA images were acquired through maximum intensity projection (MIP). The image for each time phase was combined into a single series.

**Table 1:** Image parameters used for Time-of-Flight (TOF), phase contrast (PC), and Time-Resolved Imaging of Contrast Kinetics (TRICKS) magnetic resonance angiography sequences.

Parameters	TOF	PC	TRICKS
Plane	Transverse	Sagittal	Dorsal
Acquisition time	11 min 4 s	5 min 10 s	2 min 44 s
TR (msec)	24	20.5	4.3
TE (msec)	2.9	5.8	1.5
FA (degrees)	25	10	15
FOV (mm)	180 × 158	180 × 162	280 × 280
Matrix	288 × 224	256 × 160	320 × 224
Voxel size (mm)	0.6 × 0.8 × 0.6	0.7 × 1.1 × 0.8	0.9 × 1.2 × 1.6
Bandwidth (Mhz)	22.7	20.83	62.5
Fat suppression	Yes	No	No
Thickness	0.6	0.8	1.6
NEX	1	0.63	1

TR; repetition time, TE; echo time, FA; flip angle, FOV; field of view, NEX; number of excitations.

### 2.4 MRA image analysis

All MRA images were sent to a picture archiving and communication system (Infinitt PACS; Infinitt Healthcare, Seoul, South Korea) for analysis. In the TOF and TRICKS MRA, both arteriogram and venogram were assessed, while in the PC MRA, only the venogram were evaluated. The vessels evaluated included seven arteries: the basilar artery, rostral cerebral artery, middle cerebral artery, caudal cerebral artery, rostral cerebellar artery, caudal communicating artery, and internal carotid artery. The venous evaluation was conducted in six dural venous sinuses and veins: the dorsal sagittal sinus, transverse sinus, temporal sinus, sigmoid sinus, straight sinus and dorsal cerebral vein.

### 2.5 Quantitative assessment of MRA

**Figure 1:**
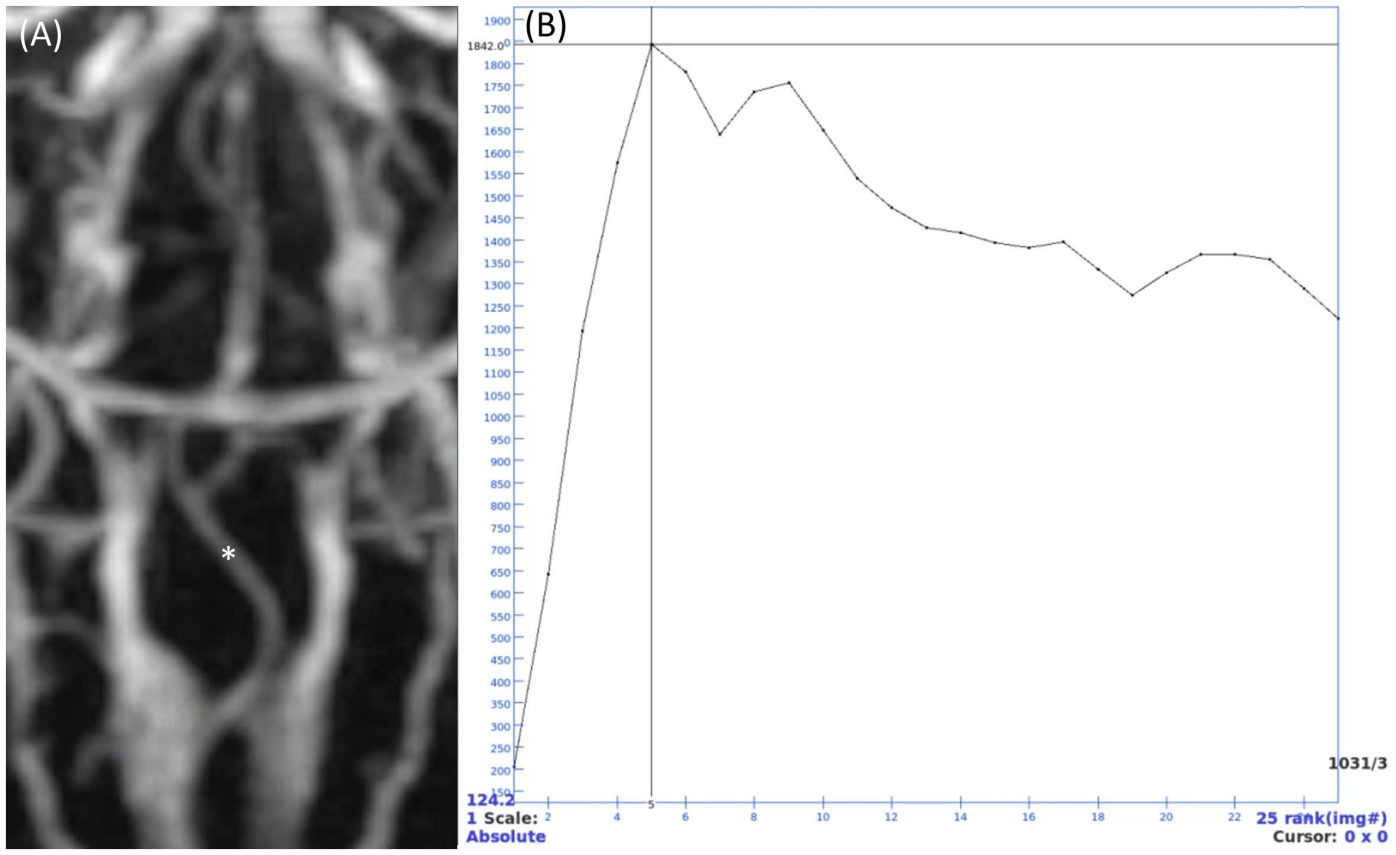
Time-resolved imaging of contrast kinetics (TRICKS) maximum intensity projection (MIP) series and time intensity curve of a basilar artery. **(A)** TRICKS MIP series from the 5th temporal phase, which showed the highest signal intensity in the basilar artery. **(B)** Intensity curve showing the signal changes of a point (indicated by asterisk in panel **A**) in basilar artery. Quantitative analysis was performed using the 5th phase, where the signal intensity peaked at 1842. After the peak intensity, the intensity gradually decreased, showing a gentle slope after the 12th phase and maintaining an intensity of approximately 1,400.

The quantitative assessment included calculating the signal-to-noise ratio (SNR) and contrast-to-noise ratio (CNR) values for the vessels observed in the three MRA sequences. The evaluations were performed by one investigator (H.S.H), a PhD candidate in veterinary medical imaging with extensive training and 10 years of experience in advanced imaging techniques. The evaluation parameters, including the selection of regions of interest (ROIs) and measurement methods, were discussed and finalized under the supervision of a diplomate of the Korean College of Veterinary Medical Imaging (J.H.C) to ensure consistency and accuracy. For TOF and PC MRA, SNR and CNR were evaluated on maximum intensity projection (MIP) images. In TRICKS MRA, due to the difficulty of accurate distinguishing between the arterial and venous phases at each time point, intensity graphs were generated for the basilar artery and transverse sinus using ROIs placed on each vessel in TRICKS MIP series. The phase with the highest intensity for the basilar artery was defined as the arterial phase, and the seven arteries were assessed by scrolling through the individual slices in this phase (Figure 1). Similarly, the phase with the highest intensity for the transverse sinus was defined as the venous phase, and the six veins were evaluated.

**Figure 2:**
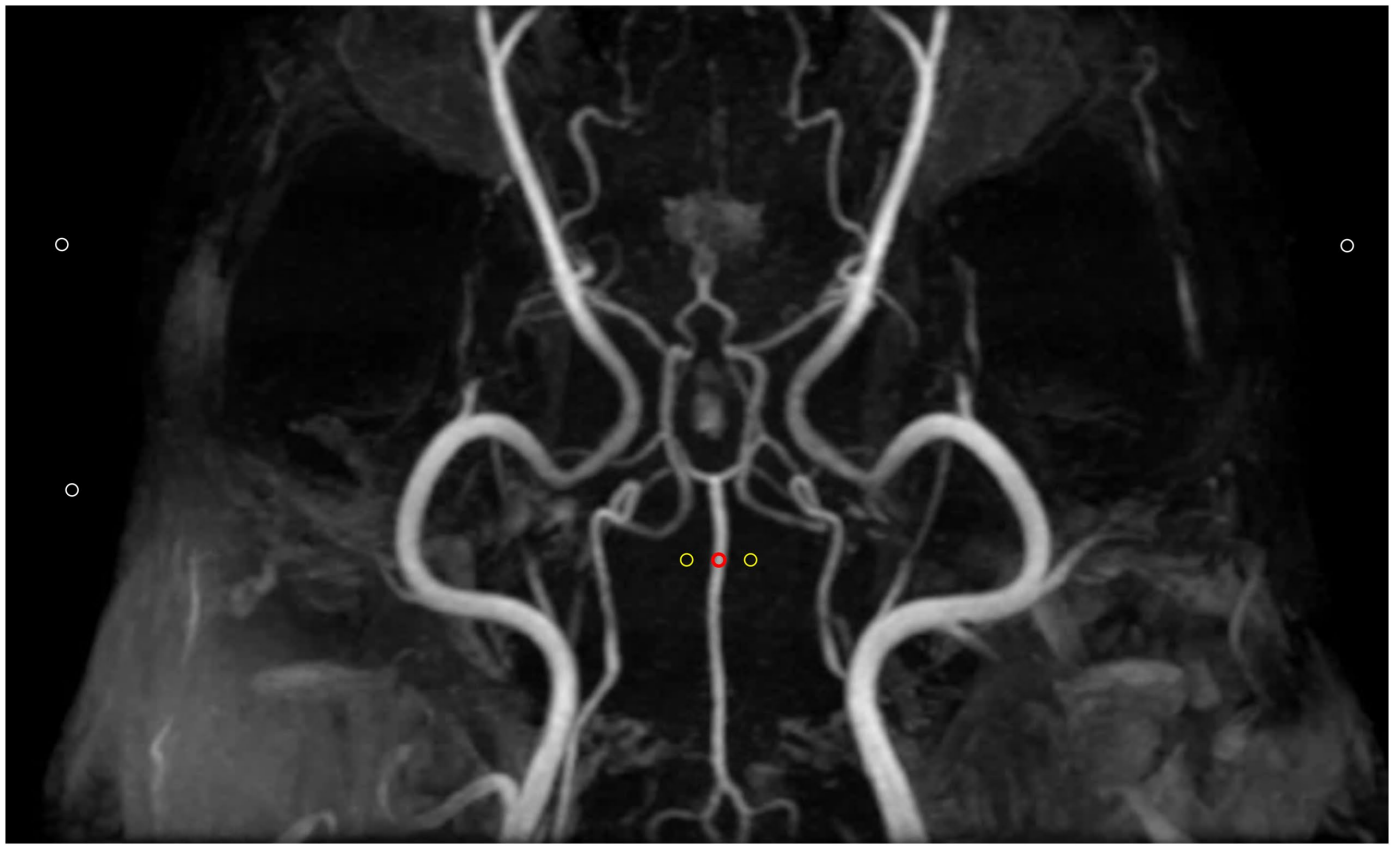
Method for defining regions of interest (ROIs) for quantitative assessment of image quality in Time-of-flight maximum intensity projection image. Three types of ROIs were used: the first ROI (red circle) was placed over a clearly visible vessel to measure vascular signal intensity. Two additional ROIs (yellow circles) with the same size were positioned in adjacent empty regions to determine background signals. Finally, three ROIs (white circles) were placed in air to measure background noise. Contrast-to-noise ratio was calculated by subtracting the mean background signal (yellow circles) from the vascular signal intensity (red circle) and then dividing by the standard deviation of the background noise (white circles). Signal-to-noise ratio was calculated as the ratio of the vascular signal intensity (red circle) to the standard deviation of the background noise (while circles).

In each MRA sequence, the signal intensity of each vessel was measured by placing a circular ROI over the vessel, ensuring that the ROI remained within the vessel boundaries and did not extend beyond the size of the vessel. For bilateral vessels, the side with the clearer visualization was selected for measurement. In cases where a vessel appeared hypoplastic, the ROI was placed over to the visible part of the vessel. If a vessel was entirely absent, the ROI was positioned in the expected anatomical location of the vessel, where it would normally be located. To account for background signal, two additional ROIs of the same size were positioned in adjacent regions devoid of visible structures near the original ROI. The average signal intensity from these two background ROIs was subtracted from the signal intensity of the vessel to calculate the CNRs. To assess background noise, three separate ROIs of equal size, were positioned in air regions, as background noise is not uniform in parallel imaging (Figure 2) (20–24). Then, for each MRA sequence, SNR and CNR for each vessel were calculated using the following formulas:


SNR=Signalintensityofthevessel/Standarddeviationofbackgroundnoise

$$\begin{array}{c} CNR=\left(\mathrm{Signal}\;\mathrm{intensity}\;\mathrm{of}\;\mathrm{the}\;\mathrm{vessel}-\mathrm{Mean}\;\mathrm{background}\;\mathrm{signal}\right)/\\ \mathrm{Standard}\;\mathrm{deviation}\;\mathrm{of}\;\mathrm{background}\;\mathrm{noise} \end{array}$$

### 2.6 Qualitative assessment of MRA

The image quality of the three MRA techniques were evaluated using sliding coronal MIP reconstructions for TOF and PC-MRA, and a single slice for TRICKS MRA. Each acquisition was independently assessed by two blinded veterinarians (H.S.H. and K.S.Y.), both PhD candidates in veterinary medical imaging. The visualization of the vessels was assessed qualitatively based on the delineation of vessel borders, homogeneity of vascular signal, and visualization of complete symmetric vessels. The assessment was conducted from seven arteries and six venous sinuses and veins separately and scored on a 4- point scale: 4 (excellent) = sharp and complete vessel borders, homogenous signal without flow artifacts, and symmetric vessel visualization, 3 (good) = slight irregularities in vessel borders, small gap, homogenous signal with slight flow artifacts, and asymmetrical but clearly visible vessels, 2 (acceptable) = irregular vessel borders, inhomogeneous signal, and incomplete, bilateral visualization, and 1 (not diagnostic) = indistinct vessel borders or unilateral visualization of bilateral vessels (25).

### 2.7 Statistical analysis

Statistical analysis was performed using SPSS for Windows (version 29.0; SPSS Inc., Chicago, IL, USA) and R (version 4.4.1, 2024) by a data analyst (L.C.M.) with a background in statistics.

The SNR, CNR, and the qualitative assessment in the visualization of the vessels of the three MRA sequences were compared using the non-parametric Friedman test, and the Wilcoxon signed rank test was used to compare the pairs of sequences when a statistically significant difference was observed. The interobserver agreement for the image quality of the arterial and venous structures was assessed with a kappa correlation test (*p* value <0.05) (26).

## 3 Results

All dogs successfully underwent the MRA sequences without any complications, such as adverse reactions to anesthesia and contrast agent administration. When comparing the acquisition times across different sequences, TOF had the longest acquisition time, followed by PC MRA, while TRICKS had the shortest acquisition time.

### 3.1 Quantitative assessment of image quality

In the quantitative assessment, no vessels appeared hypoplastic or absent in the arteriogram of TOF. However, in the venogram of TOF, vessels appeared hypoplastic in 9 cases (transverse sinus: 4, temporal sinus: 2, sigmoid sinus: 2, straight sinus: 1), and complete absence was observed in 7 cases (temporal sinus: 1, sigmoid sinus: 1, straight sinus: 1, dorsal cerebral vein: 4). In the venogram of PC MRA, vessels appeared hypoplastic in 11 cases (transverse sinus: 2, temporal sinus: 3, sigmoid sinus: 3, dorsal cerebral vein: 2), and complete absence was observed in 2 cases (sigmoid sinus: 1, straight sinus: 1).

SNR and CNR values for seven arteries and six venous structures are presented in Tables 2, 3. TOF sequence showed significantly higher SNR in most arteries, including the basilar, rostral cerebral, caudal cerebral, and caudal communicating arteries compared to TRICKS. Additionally, TOF sequence had significantly higher CNR in the basilar artery, whereas TRICKS showed significantly higher CNR in the internal carotid artery. In the venous structures, TRICKS had the highest SNR in the transverse and temporal sinuses compared to TOF and PC. TRICKS also was comparable to TOF in the sigmoid sinus, straight sinus, and dorsal cerebral vein, their images better than in PC. TRICKS demonstrated the highest CNR in most veins, including the transverse, temporal, sigmoid, dorsal cerebral, and straight sinuses.

**Table 2:** Comparison of signal-to-noise ratios (SNR) and contrast-to-noise ratios (CNR) between Time-of-Flight (TOF) and Time-Resolved Imaging of Contrast Kinetics (TRICKS) sequences in each of the seven arteries and the whole arteries.

Evaluated factor	Evaluated arteries	TOF (*n* = 5)	TRICKS (*n* = 5)	*p* values
SNR	Basilar artery	395 (323–505)^*^	169 (92–204)	0.04
	Rostral cerebral artery	300 (221–430)^*^	217 (66–234)	0.04
	Middle cerebral artery	235 (183–370)	173 (133–198)	0.01
	Caudal cerebral artery	227 (171–317)^*^	78 (74–149)	0.04
	Rostral cerebellar artery	212 (156–279)	104 (73–158)	0.08
	Caudal communicating artery	295 (211–433)^*^	132 (71–179)	0.04
	Internal carotid artery	297 (218–409)	216 (149–501)	0.35
	Whole arteries	279 (156–505)^*^	158 (66–501)	0.00
CNR	Basilar artery	257.83 (218.25–360)^*^	151.55 (85.62–186.21)	0.04
	Rostral cerebral artery	142.33 (113.03–267.59)	202 (57.65–214.3)	0.89
	Middle cerebral artery	98 (57.58–210.52)	142.86 (124.91–179.86)	0.50
	Caudal cerebral artery	64.54 (63.67–159.31)	65.65 (45.59–116.9)	0.35
	Rostral cerebellar artery	64.67 (34.85–122.59)	85.51 (59.91–138.29)	0.50
	Caudal communicating artery	176.52 (91.06–285.34)	111.73 (57.12–142.79)	0.08
	Internal carotid artery	142.22 (100.76–256.03)	199.3 (139.91–482.93)^*^	0.04
	Whole arteries	142.22 (34.85–360)	138.56 (45.59–482.93)	0.27

All data are presented as median (ranges).

* *p* value < 0.05.

**Table 3:** Comparison of signal-to-noise ratios (SNR) and contrast-to-noise ratios (CNR) among Time-of-Flight (TOF), Phase contrast (PC), and Time-Resolved Imaging of Contrast Kinetics (TRICKS) sequences in each of the six venous structures and the whole veins.

Evaluated factor	Evaluated veins	TOF (*n* = 5)	PC (*n* = 5)	TRICKS (*n* = 5)
SNR	Dorsal sagittal sinus	219 (161-278)^a^	84 (52-119)^b^	138 (23-244)^a, b^
	Transverse sinus	144 (99-155)^a^	106 (77-129)^b^	441 (232-560)^c^
	Temporal sinus	136 (55-177)^a^	136 (87-173)^a^	439 (265-614)^b^
	Sigmoid sinus	123 (53-181)^a^	37 (30-52)^b^	157 (117-444)^a^
	Straight sinus	105 (57-157)^a^	32 (18-52)^b^	177 (71-387)^a^
	Dorsal cerebral vein	83 (48-204)^a^	36 (18-38)^b^	104 (90-174)^a^
	Whole veins	133 (48-278)^a^	52 (18-173)^b^	182 (23-614)^c^
CNR	Dorsal sagittal sinus	141.1 (81.9–179.51)^a^	68.65 (40.61–103.91)^b^	125.05 (13.95–207.42)^a, b^
	Transverse sinus	65 (46.15–91.69)^a^	89.29 (63.54–115.65)^a^	418.4 (211.6–531.93)^b^
	Temporal sinus	57.5 (6.62–83.78)^a^	119.91 (77.03–156.5)^b^	419.73 (256.05–593.13)^a^
	Sigmoid sinus	42.4 (0.2–75.73)^a^	21.3 (17.38–37.31)^a^	147.59 (107.9–407.83)^b^
	Straight sinus	31.09 (8.58–57.91)^a^	18.89 (8.69–38.30)^b^	164.76 (60.85–366.68)^c^
	Dorsal cerebral vein	7.32 (−1.28–131.5)^a^	21.79 (4.81–25.29)^a^	96.45 (77.95–154.34)^b^
	Whole veins	57.16 (−1.28–179.51)^a^	39.45 (4.81–156.50)^a^	165.76 (13.95–593.13)^b^

All data are presented as median (ranges).

^a–c^ Within a row, values with the same superscripted letters indicate statistically significant differences between the two groups (*p* < 0.05).

**Figure 3:**
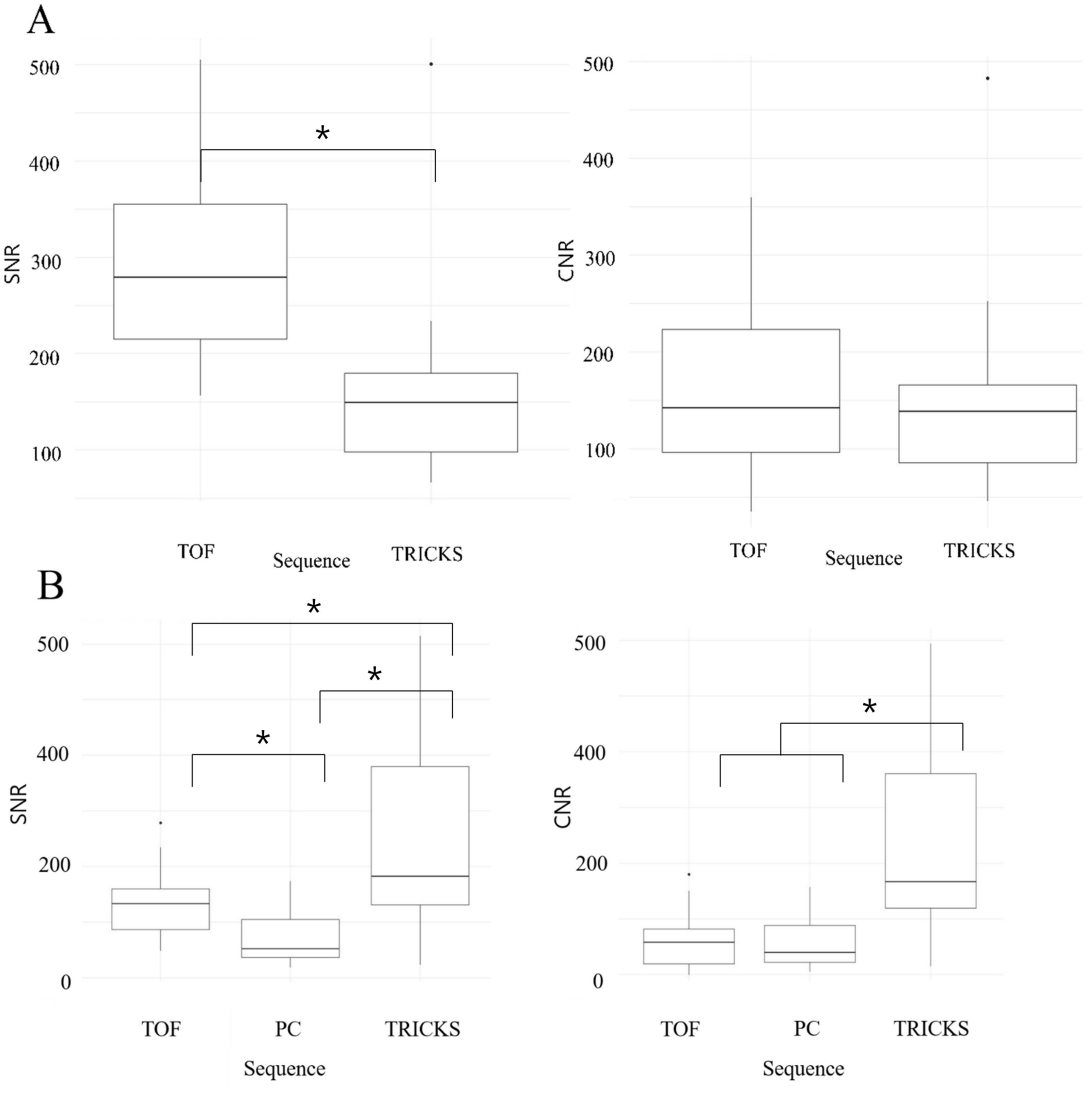
Box and whisker plots comparing the signal-to-noise ratios (SNRs) and contrast-to-noise ratios (CNRs) across different magnetic resonance angiography (MRA) sequences for cerebral arteries and veins. **(A)** SNRs and CNRs of Time-of-Flight (TOF) and Time-Resolved Imaging of Contrast Kinetics (TRICKS) MRA sequences for cerebral arteries, including basilar artery, rostral cerebral artery, middle cerebral artery, caudal cerebral artery, rostral cerebellar artery, caudal communicating artery, and internal carotid artery. TOF showed higher SNRs compared to TRICKS, with statistically significant differences (*p < 0.05). **(B)** SNRs and CNRs of TOF, Phase Contrast (PC), and TRICKS MRA sequences for cerebral veins, including dorsal sagittal sinus, transverse sinus, temporal sinus, sigmoid sinus, straight sinus, and dorsal cerebral vein. TRICKS achieved the highest SNRs and CNRs among the three sequences, with statistically significant differences (*p < 0.05).

When comparing the combined SNR and CNR values across all arteries between TOF and TRICKS MRA sequences, TOF demonstrated higher SNR than TRICKS, though no significant difference in CNR were found between the two sequences. In combined SNR and CNR values across all venous structures among the three MRA sequences, TRICKS demonstrated the highest combined SNR, followed by TOF and PC. The CNR value was also highest in TRICKS compared to the other two sequences. These results are graphically represented in Figure 3.

### 3.2 Qualitative assessment of image quality

**Figure 4:**
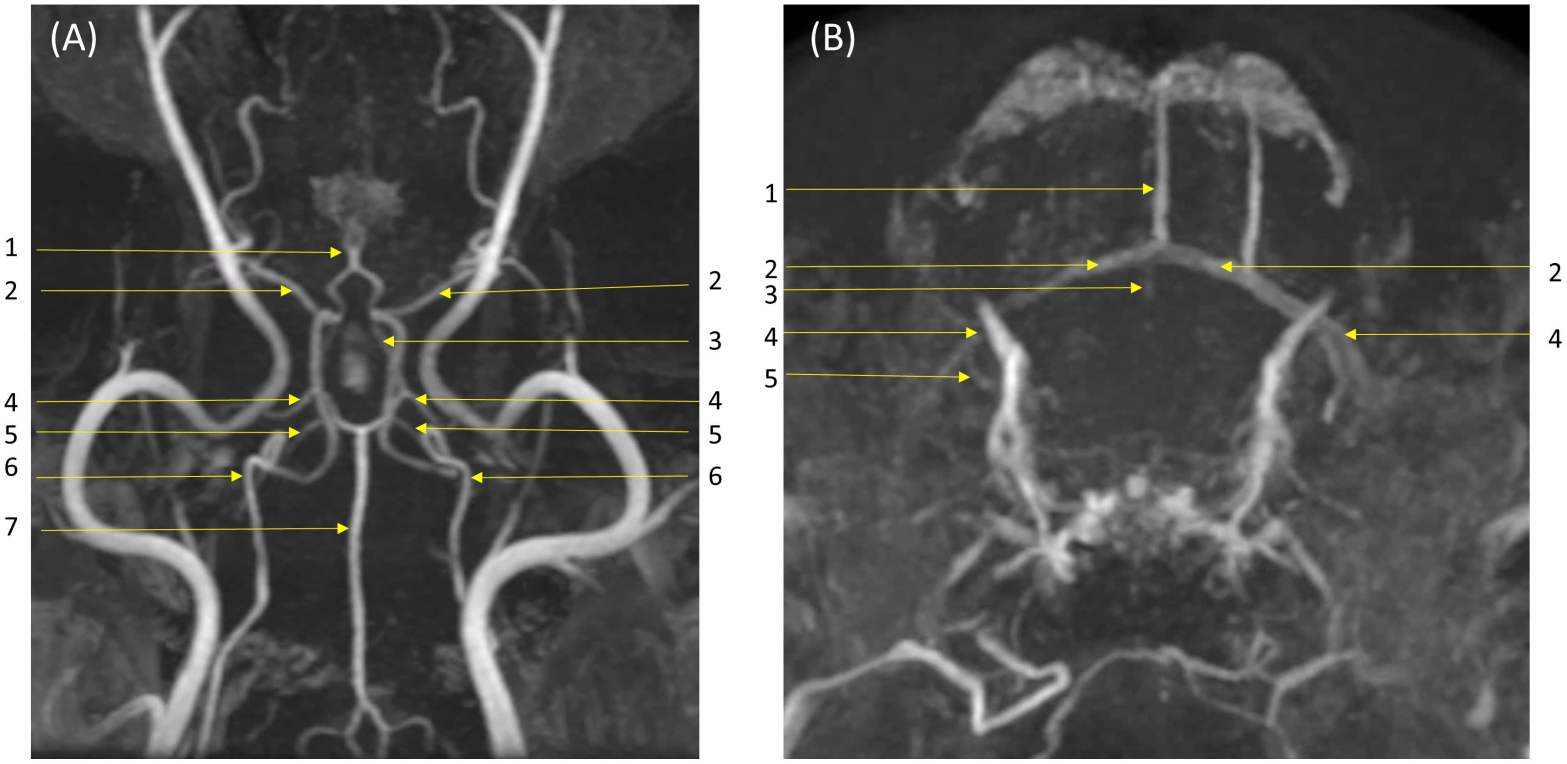
Visibility of brain vessels in Time-of-Flight (TOF) maximum-intensity projection images. **(A)** Arteriogram in dorsal and transverse orientations. Major arteries were depicted clearly to an acceptable or higher level: (1) rostral cerebral artery; (2) middle cerebral artery; (3) caudal communicating artery; (4) caudal cerebral artery; (5) rostral cerebellar artery; (6) internal carotid artery; (7) basilar artery. **(B)** Venograms in dorsal and transverse orientations. Visibility of veins is generally low due to in-plane saturation artifact: however, the dorsal sagittal sinus is clearly visible and less affected by artifact. Labeled veins: (1) dorsal sagittal sinus; (2) transverse sinus; (3) straight sinus; (4) temporal sinus; (5) sigmoid sinus. The dorsal cerebral vein is not visualized.

**Figure 5:**
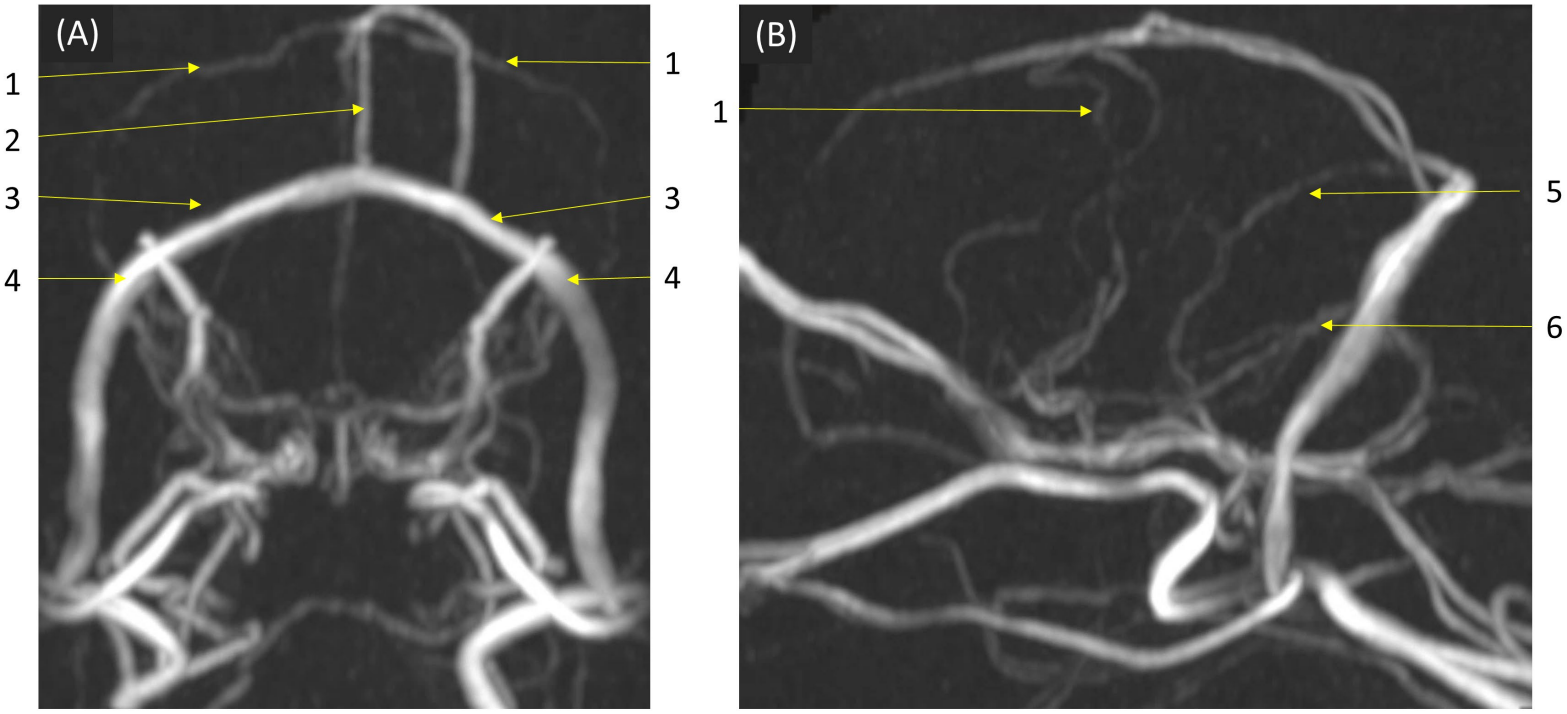
Visibility of brain veins in Phase Contrast (PC) MRA maximum-intensity projection images. This sequence provided acceptable visibility for venous structures, including the dorsal sagittal sinus, straight sinus, and dorsal cerebral vein, demonstrating its effectiveness in venous imaging. **(A)** Transverse and **(B)** sagittal images, at the level of the confluence of the dorsal, sagittal, transverse, and straight sinuses. Labeled venous structures are: (1) dorsal cerebral vein; (2) dorsal sagittal sinus; (3) transverse sinus; (4) temporal sinus; (5) straight sinus; (6) sigmoid sinus.

**Figure 6:**
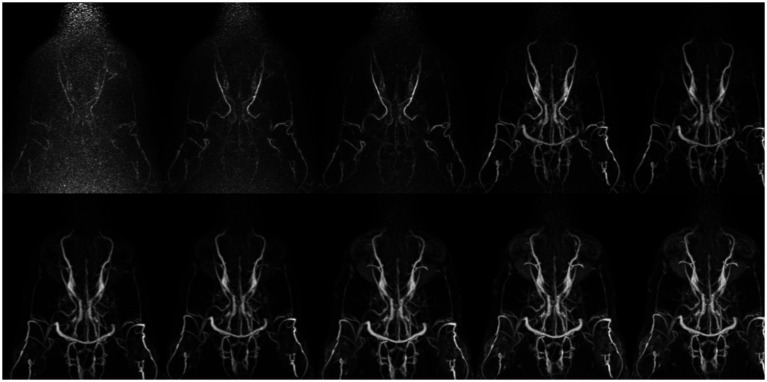
Collapsed dorsal images of 10 contrast-enhanced temporal phases of Time-Resolved Imaging of Contrast Kinetics (TRICKS) sequences. The images show that sufficient contrast filling of the cerebral dural venous sinuses is obtained before the sixth temporal phase. The temporal resolution was 5.9 s for each phase. TRICKS demonstrated generally excellent visibility of both arteriograms and venograms; however, clear distinction between the arterial and venous phases was challenging due to overlapping contrast enhancement.

In qualitative assessments, TOF MRA demonstrated acceptable or higher vascular visibility in most of brain arteries (Figure 4A); though visibility in venogram generally low for many veins due to in-plane saturation artifacts, except for the dorsal sagittal sinus (Figure 4B). Specifically, the sigmoid sinus and dorsal cerebral vein were challenging to observe in any dogs. PC MRA provided acceptable visibility for the dorsal sagittal sinus, straight sinus, and dorsal cerebral vein (Figure 5); however, it exhibited considerable inter-animal variability in visibility for the transverse sinus, temporal sinus, and sigmoid sinus. Moreover, the basilar artery and internal carotid artery were observed alongside venous structures in the venogram, resulting in arterial signal interference that poses challenges in distinguishing exclusively venous structures. TRICKS demonstrated generally excellent visibility in both arteriograms and venograms (Figure 6); however, the image quality was somewhat lower for the caudal communicating artery and the dorsal cerebral vein. Furthermore, there was no phase exclusively representing the arterial phase without venous contamination, making it difficult to clearly differentiate between arterial and venous phases. The phases at which vascular signals peaked after contrast administration varied, with arterial peaks observed between phases 5 and 8, and venous peaks between phases 7 and 8. Each MRA sequence was assessed qualitatively about delineation of vessel borders, homogeneity of vascular signal, and visualization of complete symmetric vessels in Tables 4, 5. Rostral cerebral artery, caudal cerebral artery, rostral cerebellar artery, and caudal communicating artery were significantly better visualized by TOF MRA compared with TRICKS. There was no significant difference in visualization of the middle cerebral artery and internal carotid artery between the TOF and TRICKS sequences. All venous structures were significantly better visualized with TRICKS MRA compared to TOF MRA. The transverse sinus, temporal sinus, sigmoid sinus, and straight sinus were better visualized with TRICKS MRA compared to PC MRA, except for the dorsal cerebral vein, which was better visualized with PC MRA than TRICKS MRA. The dorsal sagittal sinus, straight sinus, and dorsal cerebral vein were better visualized with PC MRA compared to TOF MRA, while there was no significant difference between the two sequences in the visualization of the transverse sinus, temporal sinus, and sigmoid sinus.

**Table 4:** Qualitative assessments about delineation of vessel borders, homogeneity of vascular signal, and visualization of complete symmetric vessels between Time-of-Flight (TOF) and Time-Resolved Imaging of Contrast Kinetics (TRICKS) magnetic resonance angiography (MRA) sequences in arteries.

Vessel score of arteries	TOF (*n* = 5)	TRICKS (*n* = 5)	*p*-value
Basilar artery	3.00 ± 0	3.00 ± 0	N/A
Rostral cerebral artery	2.70 ± 0.48^*^	1.90 ± 0.31	0.008
Middle cerebral artery	2.40 ± 0.69	2.00 ± 0	0.102
Caudal cerebral artery	2.70 ± 0.48^*^	2.00 ± 0	0.008
Rostral cerebellar artery	2.80 ± 0.42^*^	1.9 ± 0.31	0.005
Caudal communicating artery	2.90 ± 0.31^*^	1.00 ± 0	0.002
Internal carotid artery	2.30 ± 0.67	2.00 ± 0	0.180

*Statistically significant difference with the non-parametric Friedman test (*p* < 0.05) and *post-hoc* test using the Wilcoxon signed rank.

N/A, not applicable.

**Table 5:** Qualitative assessments about delineation of vessel borders, homogeneity of vascular signal, and visualization of complete symmetric vessels among Time-of-Flight (TOF), Phase contrast (PC) and Time-Resolved Imaging of Contrast Kinetics (TRICKS) magnetic resonance angiography (MRA) sequences in venous structures and veins.

Vessel score of veins	TOF (*n* = 5)	PC (*n* = 5)	TRICKS (*n* = 5)
Dorsal sagittal sinus	1.10 ± 0.31^a^	3.00^b^	2.90 ± 0.31^b^
Transverse sinus	0.70 ± 0.82^a^	1.50 ± 1.17^a^	2.70 ± 0.48^b^
Temporal sinus	0.60 ± 0.84^a^	1.40 ± 1.07^a^	2.30 ± 0.67^b^
Sigmoid sinus	0^a^	0.50 ± 0.70^a^	1.90 ± 0.73^b^
Straight sinus	0.70 ± 0.48^a^	1.50 ± 0.70^b^	2.20 ± 0.42^c^
Dorsal cerebral vein	0^a^	1.70 ± 0.48^b^	1.00^a^

Data are expressed as mean ± standard deviation.

^a–c^ Within a row, values with the same superscripted letters indicate statistically significant differences between the two groups (*p* < 0.05).

Interobserver agreement was strong, with average weighted kappa values of 0.845 for TOF MRA, 0.821 for PC MRA, and 0.876 for TRICKS MRA, indicating substantial to almost perfect reliability across evaluators.

## 4 Discussion

This study evaluated the effectiveness of TOF, PC, and TRICKS MRA sequences for imaging cerebral arteries and veins in normal dogs. TOF MRA provided excellent visualization for major cerebral arteries with high SNR and CNR values, especially in the rostral cerebral, caudal cerebral, and rostral cerebellar arteries. TRICKS MRA, however, was better than TOF in the internal carotid artery, likely due to its ability to minimize artifacts from flow direction changes. For venous imaging, TRICKS achieved the highest SNR and CNR across most veins and PC MRA was optimal only in the dorsal sagittal sinus. Each MRA sequence provided specific advantages based on the vascular structures of interest in canine brain.

TOF provided better visualization with higher SNR and higher CNR than TRICKS in most arteries, particularly in the rostral and caudal cerebral arteries and the rostral cerebellar artery and was comparable for some arteries when compared to TRICKS. These attributes make TOF advantageous for visualizing major cerebral arteries, as demonstrated in the previous studies using TOF for canine cerebral artery imaging (8–11, 27). In those studies, the TOF sequence demonstrated excellent SNR and CNR, providing high-resolution visualization of major arteries. However, human studies emphasized the consideration of the limitations in TOF such as signal loss due to slow flow or flow parallel to the scan plane when using this sequence (28). Another canine study using TOF imaging in 39 dogs with idiopathic epilepsy, the rostral cerebellar artery, caudal communicating artery, and internal carotid artery were not clearly visualized, suggesting that alternative diagnostic imaging techniques, such as contrast-enhanced MRA. Similarly, in our study, TRICKS showed higher SNR and CNR in the internal carotid artery, likely due to the unique variability in internal carotid artery diameter between proximal and distal regions in dogs and intravoxel phase dispersion artifacts in areas with kinks or loops in the internal carotid artery affecting TOF images in regions with vessel curvature (7). As a CE-MRA technique, TRICKS provides consistent signal intensity regardless of blood flow direction, making it less susceptible to artifacts from flow velocity changes, a common issue in TOF (29).

In the venogram of MRA, TRICKS demonstrated significantly higher SNR and CNR compared to PC MRA in most venous structures, except the dorsal sagittal sinus where PC MRA showed the highest SNR and CNR. TRICKS MRA exhibited superior SNR and CNR values compared to TOF in the temporal sinus, straight sinus, and dorsal cerebral vein. When evaluated across all veins collectively, TRICKS MRA yielded the highest SNR and CNR values and significantly higher scores in qualitative assessments than other sequences across all venous structures except for the dorsal cerebral vein. A few veterinary studies have applied TOF venograms to cases with cavernous sinus syndrome and intracranial intravascular lymphoma (30, 31); however, our study is the first to evaluate intracranial TOF venogram in normal canine brains. In our study, TOF showed lower visibility in most cerebral veins, except for the dorsal sagittal sinus which is oriented perpendicular to the plane direction, compared to other sequences, likely due to in-plane saturation artifacts and slow venous flow (32). Moreover, the intracranial venous system is a complex three-dimensional structure where blood flows in multiple directions, making it more susceptible to signal loss. TOF MRA is particularly prone to saturation effects, as it excites a thicker slab of tissue, and it is less sensitive to detecting slow blood flow. For these reasons, oblique plane techniques are sometimes used to improve vessel contrast compared to direct orientations, especially when veno-occlusive disease is suspected in human medicine (33). Therefore, 2D TOF is often preferred for MR venography due to its lower susceptibility to in-plane saturation artifacts compared to 3D TOF. Consequently, 2D TOF or 3D PC MRA is often favored for the venogram.

Although PC MRA showed significantly lower SNR values compared to TOF and TRICKS, PC MRA received higher qualitative scores for certain structures like the dorsal sagittal sinus, straight sinus, and dorsal cerebral vein. This discrepancy may be due to the better background suppression effect of PC MRA compared to TOF venogram. Although PC MRA has been used in previous veterinary studies for cerebrospinal fluid flow and abdominal vascular imaging, its application in brain studies has primarily focused on dorsal dural venous sinus variations in one canine study, which showed transverse sinus asymmetry in 58.8%, hypoplasia in 39.2% and aplasia in 23.5% (13, 34–36). Similar transverse sinus findings were observed on PC MRA in our study, such as unilateral aplasia in one dog (20%) and unilateral hypoplasia in two dogs (40%). However, these results were considered due to inappropriate velocity-encoding values, because TRICKS consistently displayed normal transverse sinus morphology in all these dogs. This result suggested the importance of optimized velocity-encoding gradients in PC MRA, especially as canine cerebral vessel velocities remain unstudied. Human studies indicate that transverse sinus blood flow velocities range from approximately 1 to 9 cm/s (32). To avoid aliasing, the optimal velocity encoding is typically set at two-thirds of the maximum average velocity. Our study used the same parameter used on the previous canine study, which set the velocity-encoding gradient at 15 cm/s to facilitate visualization of slow-velocity venous flow. However, as there have been no studies conducted on the velocity of canine cerebral vessels for determining the optimal velocity-encoding gradient, further research is required. A recent human study compared phase-contrast MRV and postgadolinium 3D-Magnetization Prepared - RApid Gradient Echo for detecting dural venous sinus thrombosis and found that the transverse dural sinus was frequently nonvisualized on PC MRV and need of additional sequence such as diffusion-weighted imaging for excluding sinus thrombosis (37, 38). Therefore, relying solely on PC MRV to diagnose dural sinus nonvisualization as aplasia or thrombosis may be insufficient and incorporating multiple imaging sequences such as DWI could enhance diagnostic accuracy. Unlike TOF and TRICKS sequences, only venograms were performed in PC MRA in this study. This decision was based on the technical characteristics of PC MRA, which is optimized for visualizing slow blood flow, such as in venous circulation, by using velocity-encoding gradients. However, PC MRA is less effective in capturing high velocity arterial blood flow due to phase dispersion and signal loss, which makes it challenge to obtain only arteriograms. Consequently, there are no established protocols or arterial-phase settings for PC MRA in animals. Therefore, to maximize its strengths and minimize potential artifacts, PC MRA was exclusively applied for venogram evaluation in this study.

The three sequences differed in acquisition times, with TOF taking the longest, followed by PC MRA, and TRICKS being the shortest. In veterinary studies, unlike human medicine, imaging is conducted under anesthesia, making the acquisition time of MRA particularly critical. Many veterinary patients have neurological issues and belong to higher anesthesia risk categories. From this perspective, TRICKS, with the shortest acquisition time among the three sequences, offers advantages in terms of anesthesia safety for patients. The rapid acquisition time achieved by the TRICKS technique is attributed to its use of keyhole imaging, a method that enhances temporal resolution by acquiring high spatial frequency information less frequently than low spatial frequency information (39–41). In previous CE-MRA studies, phase duration times were set to 13–14 s for renal studies, 12–13 s for portal venous studies, and 10 s for intracranial and extracranial vascular studies (41–44). In contrast, this study achieved a significantly shorter phase duration of approximately 5.9 s, enabling superior temporal resolution in a shorter timeframe.

However, compared to previous CE-MRA studies, the total acquisition time in our study was relatively longer at 2 min and 44 s. In previous studies, the acquisition time for renal MRA was 1 min, 65 s for portal venous studies, and 50 s for intracranial and extracranial vascular studies (42–44). The extended acquisition time in our study was intentional to allow a more prolonged observation of dynamic blood flow, as there is little research on TRICKS for cerebral angiography in dogs. As a result, the time intensity curve showed a gradual slope in blood flow signal intensity beyond the 14th phase. Therefore, limiting the phase setting to the 14th phase is expected to enable the completion of the sequence within 2 min, making the process more time-efficient while still retaining critical diagnostic information. However, despite this short phase acquisition time, it was difficult to clearly distinguish between the arterial and venous phases in cerebral vessels, unlike in portal vein and renal contrast enhanced MRA studies where such differentiation was possible. In previous contrast enhanced MRA studies of intracranial vessels with a phase acquisition time of 10 s, there were also difficulties in distinguishing between the arterial and venous phases (44). Further research is needed to determine the appropriate amount and injection rate of contrast agent and flushing saline tailored and establish the optimal protocol for canine MRA to depict only the arterial phase.

This study has some limitations. First, this study was performed in small sample size including only five normal beagle dogs. This was because the study was designed as exploratory pilot study to evaluate the feasibility and effectiveness of the MRA protocols. Further studies with larger and more diverse cohorts are needed to confirm these findings. Second, since the optimal protocols for TOF venogram, PC MRA, and TRICKS MRA have not been established in dogs, there were limitations in comparing the sequences at their maximum performance. Further research should focus on developing optimized imaging protocols to enable more accurate and consistent comparisons among MRA sequences. Thirdly, the TRICKS sequence used only single-slice evaluation rather than MIP used in TOF and PC sequences. This approach for the TRICKS sequence was chosen due to the inherent characteristics of this technique. Unlike TOF and PC MRA, which rely on static imaging for MIP reconstructions, the TRICKS technique captures dynamic, time-resolved contrast data. This dynamic nature makes it challenging to generate accurate MIP images without introducing artifacts from overlapping arterial and venous phases and obtain phases where arteries and veins were clearly differentiated. Therefore, single-slice evaluations were used to minimize potential artifacts and ensure clearer phase-specific analysis. However, this approach may have introduced limitations in visualizing the complete vascular anatomy. These limitations may have introduced inaccuracies, including the potential overestimation or underestimation of partial vascular structures.

This study quantitatively and qualitatively compared TOF, PC MRA, and TRICKS sequences for visualizing the cerebral arteries and veins in dogs. TOF showed superior SNR, CNR, and vessel visibility for cerebral arteries, and indicating TOF as the recommendation for the cerebral arteries evaluation. While TRICKS is also effective for arterial imaging with rapid acquisition, this MRA sequence is particularly recommended for venous imaging due to its higher SNR, CNR, and vessel visibility, especially when shorter acquisition times are a priority in dogs.

## Data Availability

The original contributions presented in the study are included in the article/supplementary material, further inquiries can be directed to the corresponding author.
